# Live fast, die young: neutrophils streamline their metabolism to maximize inflammation

**DOI:** 10.1128/iai.00498-24

**Published:** 2025-06-30

**Authors:** Bailey E. Holder, Callista P. Reber, Andrew J. Monteith

**Affiliations:** 1 Department of Microbiology, University of Tennessee 189504 , Knoxville, Tennessee, USA; 2 Department of Biochemistry & Cellular and Molecular Biology, University of Tennessee, Knoxville, Tennessee, USA; University of Pittsburgh, Pittsburgh, Pennsylvania, USA

**Keywords:** neutrophils, metabolism, bacterial infection, mitochondria, diabetes, systemic lupus erythematosus, glycolysis, inflammation, pentose phosphate pathway, obesity

## Abstract

Neutrophils are the most abundant leukocytes at sites of inflammation and form the front line of the innate immune response. Neutrophils have a relatively short lifespan compared to other cell types, as they have streamlined their metabolic processes to support an arsenal of antimicrobial functions to combat invading pathogens at the cost of maximizing ATP output. To elicit antimicrobial stress, neutrophils rewire their glycolytic pathways to sustain phagocytosis and the oxidative burst and modify their mitochondrial metabolism to dictate degranulation or release of neutrophil extracellular traps. While many of these effector functions are sufficient to protect the “healthy” host from infection, chronic diseases disrupting metabolic and inflammatory homeostasis render the host susceptible to more frequent and severe bacterial infections. With the growing incidence of many metabolic and autoimmune diseases, a clearer understanding of the mechanisms regulating or disrupting neutrophil antimicrobial processes is required. This review focuses on the relationship between neutrophil function and metabolism and what is known about how this impacts autoimmune and metabolic diseases and/or disorders in the case of bacterial infection.

## INTRODUCTION

Cell metabolism plays a key role in shaping immune cell differentiation and function, with each immune cell subset depending on distinct metabolic pathways. While there have been extensive studies and reviews on the concepts of immunometabolism as it relates to T cells, B cells, and macrophages, neutrophils have been often overlooked. However, recent evidence indicates that neutrophils are metabolically complex and leverage metabolic processes to support their antimicrobial functions. As such, many metabolic and autoimmune diseases correlate with an increased susceptibility to bacterial infection, suggesting that the altered inflammatory or metabolic landscape within the host disrupts neutrophil function. This review focuses on the relationship between neutrophil function and metabolism and what is known about how this impacts autoimmune and metabolic diseases/disorders in the case of bacterial infection.

## NEUTROPHILS FORM THE BACKBONE OF THE INNATE IMMUNE RESPONSE

Neutrophils are the most abundant leukocyte and one of the first immune cells at the site of infection. As the front line of the immune response, they are equipped with an arsenal of antimicrobial processes to respond to invading pathogens. Tissue resident cells produce cytokines and chemokines to facilitate the recruitment of neutrophils to the site of infection (1). In addition, microbes can cause tissue damage, leading to the production of damage-associated molecular patterns (DAMPs) that are recognized alongside pathogen-associated molecular patterns (PAMPs), by the pattern recognition receptors (PRRs) neutrophils possess (2). Neutrophils that directly engage the pathogen recognize and rapidly phagocytose invading microbes (3), attempting to kill microbes in the phagosome by producing high concentrations of reactive oxygen species (ROS) via the respiratory burst by NADPH oxidase (4). In the phagosome, NADPH oxidase converts NADPH and oxygen into superoxide anions, which rapidly dismutate into hydrogen peroxide (5, 6). This can be further converted into hypochlorous acid and other strong oxidants by myeloperoxidase (MPO) (7–9). These ROS not only cause direct oxidative stress upon microbes, but also host lipids, such as arachidonic acid (AA), can be oxidized to elicit toxic effects on phagocytosed microbes (10). While the oxidative burst serves to broadly incapacitate microbial pathogens, microbes have evolved strategies to persist within the phagosome (11). Some microbes can produce pigments like the carotenoids staphyloxanthin in *Staphylococcus aureus* and granadaene in *Streptococcus agalactiae* and anthocyanins like pyocyanin in *Pseudomonas aeruginosa*, which directly neutralize ROS stress by scavenging free radicals, thereby preventing damage (12–14). Alternatively, microbes can express enzymes like catalase (15, 16) or methionine sulfoxide reductase (17) that can decompose oxidants into less harmful products. Superoxide dismutase (SOD) has been shown to contribute to survival against NADPH oxidase-mediated killing (18), but SOD likely plays a larger role in combating oxidative stress as a result of microbial metabolic processes. Thus, neutrophils require supplementary antimicrobial processes to effectively protect the host from infection.

Neutrophils possess a plethora of antimicrobial peptides and proteins (AMPs) contained within granules to complement the oxidative burst (19). Azurophil granules contain the most toxic compounds, including MPO to augment the oxidative burst; degradative enzymes such as lysozyme, proteinase 3, cathepsin G, and neutrophil elastase (NE); and defensins that disrupt the integrity of microbial membranes (20–25). Specific granules contain some of the degradative enzymes found in azurophilic granules but are also enriched in metal-sequestering proteins like lipocalin and lactoferrin, as well as enzymes critical in degrading extracellular matrices, such as collagenase and gelatinase (26, 27). Tertiary granules are further enriched in matrix-degrading enzymes (28), and secretory granules contain a diverse array of signaling proteins and membrane receptors that are readily secreted (29, 30). These granules can directly fuse to the phagosome to kill internalized microbes or can also be released extracellularly to kill microbes in proximity to the neutrophil through a process termed “degranulation” (31). Granule release occurs sequentially based on calcium flux, with secretory granules readily released throughout the lifespan of the neutrophil and primary granules requiring the greatest stimulus for release (32, 33). However, some microbes can evade AMPs released from granules by modifying the electrostatic properties of their cell membrane to prevent the antimicrobial activity of defensins (34, 35). Bacterial molecules such as siderophores, as well as metal transporters, mitigate the effects of metal sequestration by neutrophil lactoferrin, lipocalin, and calprotectin (36, 37).

Neutrophils can also release a complex meshwork of DNA studded with AMPs called “neutrophil extracellular traps” (NETs) through two processes: suicidal NETosis and vital NET release. Suicidal NETosis was the first identified form of NET release (38), which results in rupture of the cell membrane and requires ROS production from NADPH oxidase (39–43) and/or mitochondria (44–53). The generation of ROS allows AMPs to escape from granules, where the combined activities of NE and MPO degrade histones and relax chromosomal DNA (41, 54, 55). NET release additionally requires peptidyl arginine deiminase 4 (PAD4) activity to citrullinate histones (56). This results in the conversion of positively charged arginine residues to neutral citrulline residues on histones, causing the release of negatively charged DNA from histone packaging. Pyroptosis may share many similarities with NET release in neutrophils, with some mechanisms of inflammasome signaling leading to the release of NETs (57). In one mechanism of caspase-1-dependent pyroptosis, stimulation by extracellular pathogens causes neutrophils to activate caspase-1, which cleaves gasdermin D, causing pyroptosis while also promoting NET release upon detection of intracellular pathogens (58). While there are many mechanistic commonalities, unique stimuli can cause differing signaling or transcriptional mechanisms that drive NETosis (43, 59), indicating suicidal NETosis is a broad antimicrobial process. Alternatively, vital NET release occurs rapidly, resulting in the release of NETs by exocytosis and maintaining the integrity of the cellular membrane (60, 61). While the molecular events regulating suicidal NETosis are relatively well characterized, the molecular mechanisms underlying vital NET release are less well-known. Initial studies indicate vital NET release to be an oxidant-independent process and are triggered by microbial factors (60). Recent studies indicate the involvement of type I IFN and PAD4 in promoting vital NET release (62). The release of NETs is critical to pathogen clearance by adhering to bacteria (38, 60, 63) to prevent dissemination (61, 64), eliciting direct antibacterial activity (47, 49, 50, 64, 65), and augmenting macrophage bactericidal activity (48, 49, 66). To combat the antimicrobial activity of NETs, multiple bacterial pathogens can produce nucleases that can degrade the DNA backbone of the NET structures, allowing for microbial dissemination and continued pathogenesis (64, 67). To counteract microbial nucleases, the association of host proteins, such as platelet factor 4 (CXCL4), may fortify NET fibers from bacterial degradation and augment NET antimicrobial activity (68–70), but this mechanism has not been fully addressed during infection. Alternatively, too much NETosis can induce collateral damage to host tissues during sepsis (71, 72) and support the growth of biofilms (73). While neutrophils possess a vast array of antimicrobial stresses that can be sufficient to clear the infection, the outcome of these neutrophil–microbe interactions is frequently decided by the metabolic and inflammatory state of the host.

## GLYCOLYSIS IS CENTRAL TO FUELING NEUTROPHIL ANTIMICROBIAL PROCESSES

Cell metabolism plays a key role in shaping immune cell differentiation and function, with each immune cell subset depending on distinct metabolic pathways. Although historically deemed metabolically simple due to their short lifespans (74), recent evidence demonstrates neutrophils to be metabolically complex. Neutrophils have a limited mitochondrial biomass (75) and rely mainly on glycolysis to generate the ATP necessary to maintain energy homeostasis (76–78), as inhibiting mitochondrial respiration has little effect on ATP levels (79). As such, neutrophils express glucose transporters (GLUT)1, GLUT3, and GLUT4 (80). Upon activation, neutrophils strongly upregulate GLUT1 expression (76, 80, 81), coinciding with increased glucose uptake (76, 82), which provides an essential carbon source for glycolysis but also rapidly depletes the environment, starving invading microbes of glucose (76, 82). In fact, depletion of glucose totally impairs most effector function (76, 77, 81, 83, 84), demonstrating glucose as an essential carbon source for neutrophils. Neutrophils also store glycogen (84, 85), which can get converted to glucose through glycogenolysis in low glucose environments (86). Proper glycogen cycling is necessary for neutrophil survival (87), resulting in a tenfold accumulation of glycogen during inflammation (85, 88), which may enhance neutrophil persistence within glucose-deplete environments during infection. However, if neutrophils can store and utilize glycogen, then this would seemingly be at odds with prior *in vitro* studies showing that exogenous glucose is required for neutrophil effector functions. One possible explanation is that the isolation process, which typically occurs through density centrifugation using glucose-deplete media, may deplete neutrophils of their intracellular glycogen reserves, causing them to depend more on extracellular glucose in the culture. Alternatively, neutrophils isolated from the bone marrow may not have maximized their glycogen stores yet, as they have not reached the blood, which contains high concentrations of available glucose. In either of these scenarios, this would argue resting neutrophils in glucose-rich media prior to assessing effector functions, so the neutrophils can maximize their glycogen reserves.

**Fig 1:**
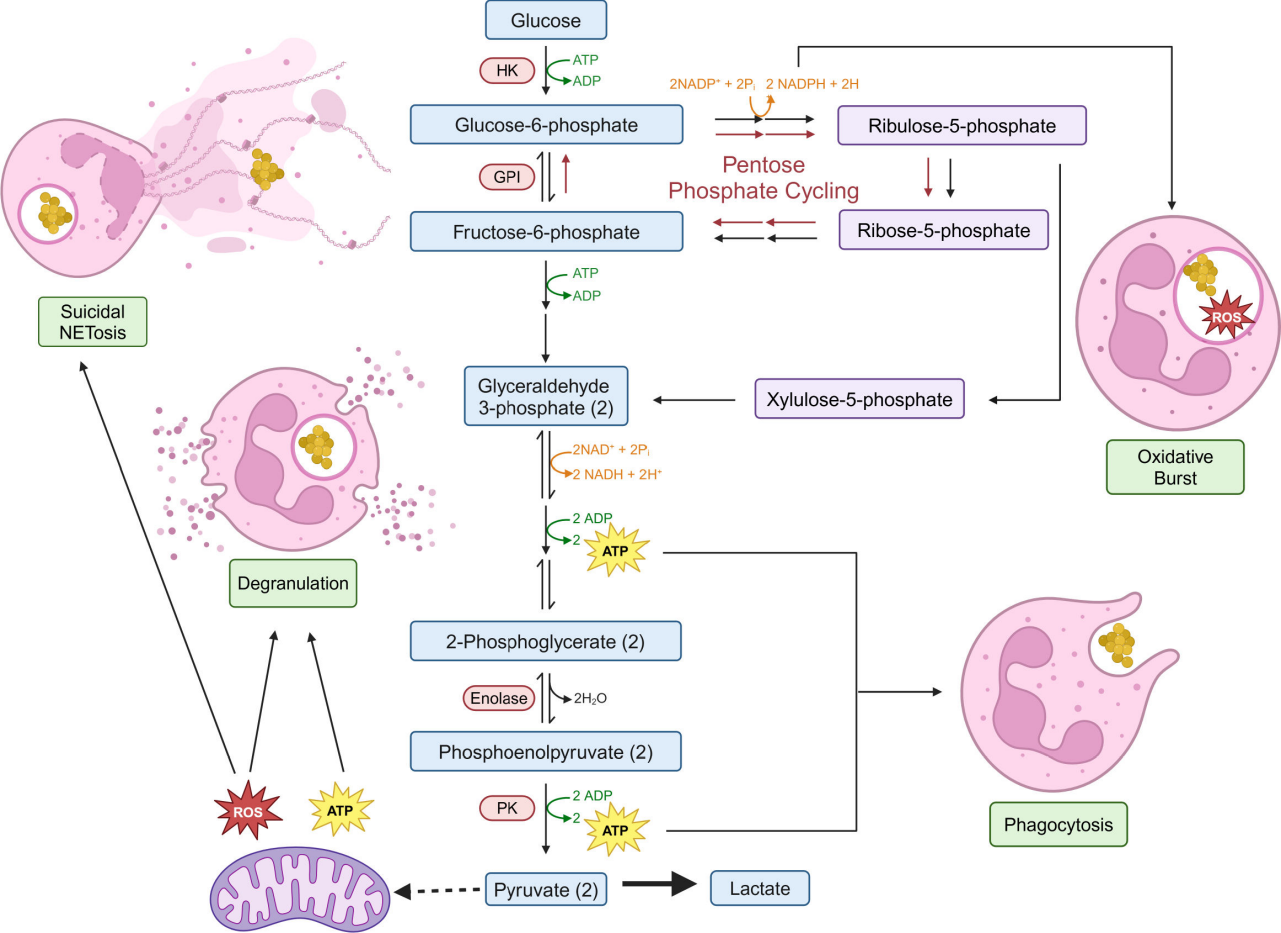
Metabolism shapes neutrophil effector functions. Upon activation, glycolytic flux is important for generating the required ATP for chemotaxis and phagocytosis of microbes. In addition, glucose flux is diverted into the pentose phosphate pathway (PPP), which produces NADPH to fuel the oxidative burst via NADPH oxidase. Neutrophils can cycle carbon through PPP up to three times through a process termed “pentose phosphate cycling” (red arrows) to maximize the production of NADPH at the cost of generating downstream pyruvate and ATP. Glycolysis largely results in the production of lactate as a metabolic byproduct rather than fluxing into mitochondria. Instead of acting as the “powerhouse of the cell”, mitochondrial-derived ROS and ATP drive neutrophil effector functions, indicating that mitochondria play unique roles in regulating neutrophil responses. Abbreviations: HK, hexokinase; GPI, phosphoglucose isomerase; PK, pyruvate kinase. Figure created with BioRender (BioRender.com).

The first step of glycolysis requires hexokinases to rapidly phosphorylate glucose into glucose-6-phosphate (G6P), preventing it from leaving the cell (Fig. 1). From here, a series of reactions converts G6P into pyruvate, which is primarily converted into lactate rather than being oxidized in the mitochondria by the tricarboxylic acid (TCA) cycle (77). Phagocytosis is an incredibly energy-intensive process (89), relying solely on ATP generated from glycolysis rather than oxidative phosphorylation (OXPHOS). As such, phagocytosis is abrogated in response to glycolysis inhibitors but insensitive to mitochondrial respiratory chain inhibitors (84, 90). Studies also found that treating neutrophils with 2-deoxy-D-glucose (2-DG) impairs phagocytosis of *S. aureus* and *Streptococcus pneumoniae* (90, 91), but treating neutrophils with sodium fluoride, an inhibitor of the glycolytic enzyme enolase, only decreases phagocytosis of *Escherichia coli*, but not *S. aureus* or *S. agalactiae* (92). The differing phenotypes may stem from their distinct sites of activity within the glycolytic pathway, with 2-DG acting early in glycolysis while sodium fluoride acting further downstream. Both inhibitors would prevent a net gain in ATP; however, inhibition of enolase would allow for glycolytic intermediates to be formed. The exact mechanism as to why differing chemical inhibitors and bacterial pathogens have inconsistent effects on phagocytosis remains unclear. Upon activation by invading pathogens, neutrophils rewire glucose flux into the pentose phosphate pathway (PPP) to support the oxidative burst (83, 93). During the oxidative pentose phosphate pathway, G6P dehydrogenase (G6PD) catalyzes the reaction of G6P to 6-phosphogluconolactone during the oxidative phase of the PPP, producing NADPH. NADPH fuels the oxidative burst by donating electrons for the reduction of molecular oxygen to superoxide, which is a starting point to produce secondary ROS. Recently, neutrophils have been shown to utilize “pentose phosphate cycling” to maximize NADPH production from G6P at the cost of producing less downstream pyruvate (93). This mechanism reverses carbon flux through the upper part of glycolysis to feed back into the pentose phosphate pathway, maximizing the amount of NADPH yielded per glucose molecule to fuel NADPH oxidase at the cost of generating ATP. Highlighting the importance of PPP, individuals with a deficiency in G6PD possess neutrophils that exhibit a diminished oxidative burst and impaired NET release, which together could account for the recurrent bacterial infections observed in these patients (94).

Glutamine is a highly abundant amino acid that is important for maintaining energy homeostasis, nucleic acid synthesis, and immune function. While glutamine has no effect on phagocytosis (95), it does enhance antimicrobial activity by enhancing the oxidative burst (95–98) but seems to have no effect on NETosis (99). In addition, activated neutrophils convert glutamine into glutamate, but rather than fueling mitochondrial processes, glutamate is used to generate glycolytic intermediates (87), which may provide an alternative mechanism for maintaining the energetic demands of phagocytosis and the oxidative burst in glucose-deplete environments.

## MITOCHONDRIA DICTATE DOWNSTREAM NEUTROPHIL EFFECTOR FUNCTIONS

**Fig 2:**
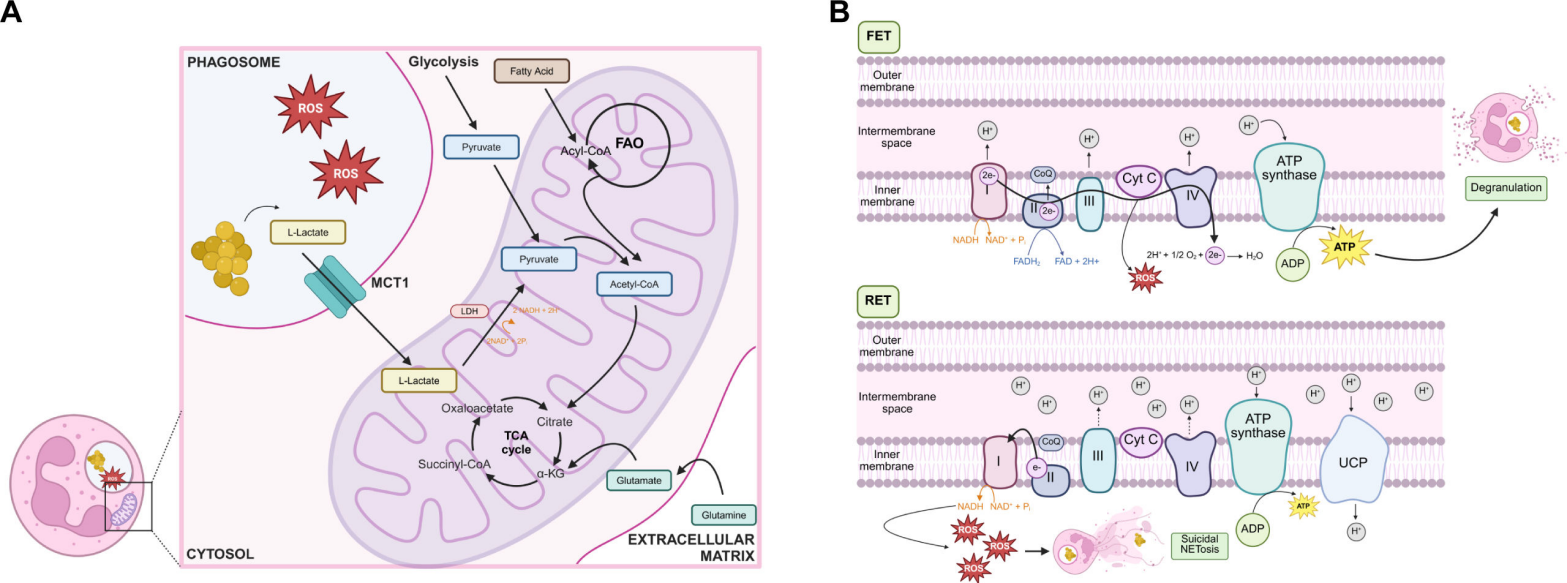
Mitochondria sense microbial persistence and dictate neutrophil effector functions. (**A**) Glycolysis, fatty acid oxidation (FAO), or glutaminolysis can fuel the TCA cycle, which may play roles in regulating neutrophil function in driving degranulation or NETosis. In addition, the mitochondria in neutrophils have also adapted to sense microbial metabolites, like L-lactate, as a metabolic danger signal consistent with persistence. The microbial lactate is assimilated into the TCA cycle, causing depletion of NAD^+^ and heightening production of mitochondrial ROS. (**B**) If carbon flux into the mitochondria is low, the electron transport chain can generate ATP during forward electron transport (FET), which acts as a mediator of degranulation. However, since the respiratory complexes in neutrophils are not efficient at shuttling electrons, increased carbon flux into the mitochondria, as during utilization of microbial lactate, overwhelms the electron transport chain and substantially heightens mitochondrial membrane potential. The increase in membrane potential causes reverse electron transport (RET), where electrons are shuttled from ubiquinol back to the respiratory complex I, thereby reducing NAD^+^ to NADH and allowing ATP synthase and uncoupling proteins (UCP) to diminish the protonmotive force. The combined depletion of NAD^+^ due to RET and lactate dehydrogenase causes significant mitochondrial ROS and triggers suicidal NETosis. Abbreviations: MCT1, monocarboxylate transporter 1; CoQ, coenzyme Q. Figure created with BioRender (BioRender.com).

Historically, the main role of mitochondria in neutrophils has been viewed as simply a means to undergo apoptosis (75, 79). Early studies even argued that mitochondria in neutrophils had minimal metabolic activity, as oxygen consumption and ATP generation were unresponsive to mitochondrial respiratory inhibitors (79, 100). However, neutrophils exhibit a complex mitochondrial network (75), express the OXPHOS complexes (101), and maintain a mitochondrial membrane potential (75, 101), suggesting that mitochondria are functional within neutrophils (Fig. 2). Despite this, mitochondrial homeostasis is unorthodox. Circulating neutrophils lack supercomplexing of the respiratory complexes. Therefore, they do not couple mitochondrial membrane potential to efficient respiration and ATP synthesis (101). Rather, the membrane potential is maintained by the transfer of electrons from glycerol-3-phosphate (G3P), a metabolic intermediate of glycolysis, where it is re-oxidized on the inner mitochondrial membrane. Subsequently, the electrons from G3P are transferred to complex III of the respiratory chain. This indicates that mitochondria are a crucial component in regulating aerobic glycolysis rather than contributing to ATP production. Across various disease and infection models, the generation of mitochondrial-derived ROS plays a critical role in driving NET release (44–53). In addition, the generation of mitochondrial ATP regulates degranulation through purinergic signaling (102). These findings could indicate that rather than acting as the “powerhouse of the cell” in neutrophils, mitochondria instead dictate downstream antimicrobial processes. Wise et al. recently demonstrated that neutrophil mitochondria detect bacterial lactate production in the phagosome as a metabolic danger signal indicating that the pathogen is persisting there (103). Rather than allowing the bacteria to replicate intracellularly, the ROS produced by the mitochondria synergizes with NADPH oxidase-derived ROS to trigger the neutrophils to undergo suicidal NETosis and eject the bacteria extracellularly entangled in a NET. The idea that mitochondria have evolved unique functions within neutrophils and are less involved in bioenergetics is unsurprising, given that neutrophils must persist and perform their antimicrobial process at the site of infection, where inflammation and microbes deplete oxygen availability (104–106).

Mitochondria also can produce ATP aerobically through fatty acid oxidation (FAO), where fatty acids enter the mitochondria through acetyl carnitine transporters and, at the end of FAO, produce acetyl-CoA molecules that can enter the TCA cycle. FAO is critical for developing neutrophils, supplying ATP for the energy-demanding process of differentiation (107, 108). In addition, FAO may be important for mature neutrophils in sustaining their effector functions in glucose-deplete environments, as fatty acid-dependent mitochondrial functions generate NADPH that allows for the neutrophils to elicit oxidative stress and prompt the release of NETs (109, 110). Exogenous non-esterified fatty acids, such as oleic acid and linoleic acid, have also been demonstrated to increase NETosis in the absence of NADPH oxidase activity (111). As many of the antimicrobial functions are controlled by metabolic processes, large-scale shifts in the metabolic homeostasis of the host may restrict the antimicrobial capacity of neutrophils and render the host susceptible to infection.

## METABOLIC AND AUTOIMMUNE DISEASES NEGATIVELY IMPACT NEUTROPHIL ANTIMICROBIAL FUNCTIONS

In recent decades, the Western diet has emerged as a result of industrialized food production. This diet is comprised of high fat and high caloric content and has led to an increased prevalence of obesity (112, 113). While obesity can lead to a plethora of complications, one of the most prevalent issues seen among obese patients is delayed wound healing and infection of chronic wounds (114). Obese individuals have an increased abundance of low-density neutrophils (LDNs), associated with a chronic state of inflammation. This subpopulation of neutrophils was initially identified in the autoimmune disease systemic lupus erythematosus (SLE) in the low-density fraction of a Ficoll density gradient (115–117), but has since then been associated with other autoimmune diseases like rheumatoid arthritis and psoriasis (118, 119). LDNs are also found in healthy individuals at low numbers, and whether they reflect immature neutrophils released from the bone marrow (120–123), overactivated neutrophils (124–127), or a distinct lineage of neutrophils (115, 117, 128) is still unclear. In addition, whether the antibacterial mechanisms, such as phagocytosis, ROS production, NETosis, and degranulation, are enhanced or diminished in response to bacterial pathogens relative to normal density neutrophils varies and may be dependent on disease context (109, 115, 129–132). Conversely, dehydration and malnutrition render mice more susceptible to bacterial infections, coinciding with altered cytokine production and neutrophil recruitment (133). Further work is required to determine the effect of diet and obesity on neutrophil development and antimicrobial functions.

Obesity is one of the most common risk factors for type 2 diabetes mellitus (T2DM). The trademark of this disease is chronic inflammation, ultimately resulting in insulin resistance and hyperglycemia (134–136). Individuals with diabetes are more prone to chronic and severe bacterial infections (137–140). Not only does excess circulating glucose heighten the virulence potential of microbial pathogens (141–143), but it can also have a direct impact on the antimicrobial capacity of immune cells. While the diabetic environment is classically considered as inflammatory, whether proinflammatory cytokines are increased or decreased varies (144–149). In addition, macrophages isolated from diabetic mice have diminished expression of GLUT1 and GLUT3, rendering them incapable of eliciting an oxidative burst (141). While macrophage antibacterial function is dampened, the effects of T2DM on neutrophils are mixed. Individuals affected by T2DM exhibit increased levels of circulating neutrophils that contribute to the constant state of inflammation experienced by these patients (150, 151). Despite the increase in circulating neutrophils, hyperglycemia dampens chemotaxis of neutrophils to sites of inflammation (152–154), impairing clearance of invading pathogens and contributing to delayed wound healing seen among T2DM patients. While neutrophils isolated from diabetic individuals display a reduced phagocytic capacity and diminished oxidative burst (144, 153, 155–157), as well as impaired degranulation (154), they express more PAD4 coinciding with heightened NETosis, which contributes to inflammation and impairs wound healing (158). Furthermore, increased deposition of double-stranded DNA, NE, and citrullinated histones is observed in individuals with T2DM, consistent with increased NET release (158, 159). Exposure to elevated concentrations of glucose *ex vivo* enhances NETosis (159, 160), which could mimic the hyperglycemic conditions *in vivo*; however, the insulin resistance developed during T2DM should prevent glucose uptake into these same cells. Conversely, it is possible that the relatively short lifespan of neutrophils or the heavy reliance on glycolysis to maintain energy homeostasis (76–78) renders neutrophils less susceptible to becoming insulin resistant and capable of utilizing the elevated levels of available glucose. Another complication of T2DM that may contribute to the dysregulation of neutrophils is the formation of advanced glycation end products (AGEs) (161–163). AGEs can aberrantly activate neutrophils, leading to a plethora of diabetic complications (162). Recently, neutrophils have been described as contributing directly to wound repair by reinforcing the extracellular matrix and aiding in reestablishing barrier function (164), but the impact of diabetes on these functions is undescribed. Collectively, these findings highlight the intricate relationship between neutrophil dysfunction and chronic inflammation in T2DM, but further work is required to untangle how the systemic shifts in metabolic homeostasis impact neutrophil effector functions in the context of infection.

The incidence and/or prevalence of many autoimmune diseases has remained steady or increased over the past few decades (165–169), and while autoimmune diseases result in large-scale shifts in immunologic homeostasis, they also can cause systemic metabolic dysregulation. Type 1 diabetes mellitus (T1DM) occurs as a result of the destruction of pancreatic beta cells by T cells, halting the production of insulin. Similar to T2DM, individuals with T1DM are more susceptible to bacterial infections (139, 140). The non-obese diabetic (NOD) mice are a well-studied murine model that spontaneously develops T1DM. While they are more susceptible to severe bacterial infections (170, 171), mild infections can attenuate the progression of diabetes due to alterations in the T cell compartment (139, 172–174). Neutrophils and NET deposition have been studied in the context of the development of T1DM (175–177), but the combined effects of autoimmunity and hyperglycemia on neutrophil effector functions during infection have not been assessed.

Other autoimmune diseases, such as rheumatoid arthritis and multiple sclerosis, present with altered neutrophil function (178–181) and an increased incidence of bacterial infections (182–185), but the links to neutrophil immunometabolism are less clear. This paragraph will focus on SLE as there has been recent work on the role of mitochondria in regulating neutrophil function. SLE is a debilitating autoimmune disease resulting from genetic and environmental components, leading to the production of autoantibodies against DNA and nuclear proteins and ultimately tissue-damaging inflammation (186–189). The source of these autoantigens is thought to derive from inefficient clearance of apoptotic debris and immune complexes (190–195). However, many of these same autoantigens can be found in granular compartments of neutrophils and NETs (46, 196–199). As such, excessive NET deposition has been observed in individuals with SLE (46, 51, 198, 200, 201), but the underlying reason for neutrophil dysregulation is still not fully resolved. Mitochondria within neutrophils play a central role in driving NETosis (44–53), and mitochondrial dysfunction has been observed in different immune cell populations in SLE (46, 50, 202–204), prior to overt immune dysregulation and tissue damage (205). While studies characterizing the metabolome of serum and leukocytes from individuals with SLE indicate broad dampening of energy-generating metabolic pathways (206–208), dyslipidemia is a common feature of SLE and involves both elevated low-density lipoproteins (LDL) and less high-density lipoproteins (HDL) (209–212). Increased abundance of oxidized LDL has been shown to enhance OXPHOS of macrophages and monocytes, leading to higher production of mitochondrial ROS (213). The effect of oxidized LDL on neutrophil mitochondrial homeostasis has not been characterized, but apoptotic debris containing immune complexes, which contain high levels of oxidized lipids, has been consistently demonstrated as a potent inducer of NET release by SLE neutrophils (46, 51, 103, 201). Despite enhanced NET release in response to immune complexes, SLE neutrophils have impaired suicidal NETosis in response to *S. aureus* (50, 103). While the underlying mechanism remains to be elucidated, dyslipidemia may disrupt metabolic homeostasis of mitochondria, causing aberrant NET formation in response to immune complexes, rendering them unresponsive to potential pathogens, and contributing to the increased incidence of severe bacterial infections observed in individuals with SLE (214–218).

## CONCLUSION

Neutrophils have historically been regarded as metabolically simplistic due to their short lifespans. However, we argue that they streamlined their metabolic pathways to augment their inflammatory output. This results in neutrophils having a “live fast, die young” lifestyle. Neutrophils rely on glycolysis to maintain energy homeostasis rather than maximizing ATP production at the mitochondria, which has three important effects (Fig. 1). First, it allows neutrophils to rapidly rewire glycolytic processes to support pentose phosphate cycling, which prioritizes the generation of NADPH to fuel NADPH oxidase over the production of ATP, while also depleting the environment of glucose to starve invading microbes. Neutrophils spend their short lifespans on the frontlines of the infection, and by restricting cellular ATP and metabolic complexity, they may limit their own survival but also the capacity of microbes to productively invade or hijack neutrophils for replication. Finally, since mitochondria are not utilized for energy homeostasis, this allows mitochondria to be adapted as “sensory organelles” that detect metabolic perturbations in the phagosome and trigger NETosis as a productive cell death pathway that removes persisting microbes from the intracellular niche (Fig. 2). The priority to generate NADPH over maximizing ATP and energy homeostasis is further supported by the Immunological Genome Project (ImmGen). The electron transport chain consumes NADH from glycolysis but can also be converted to NADPH by NADH kinase (NADK). ImmGen shows that NADK is highly expressed by all myeloid cells, but is highest of all in neutrophils, indicating that neutrophils are set up to value NADPH more than mitochondrial respiration. Similarly, ImmGen also shows that neutrophils express the highest level of G6PD among immune cells, indicating that neutrophils are better equipped to shunt glucose into the PPP rather than engaging glycolysis to generate ATP. This tailoring of metabolism to suit the physiological needs of neutrophils makes them unique, but also makes it difficult to extrapolate immunometabolism principles from more metabolically complex cells like macrophages, T cells, and B cells to understand neutrophils within the context of disease.

Metabolite availability can fluctuate temporally and spatially within the host, which means that assessing neutrophil responses *in vivo* can vary depending on when or where the infection occurs. In addition, many chronic autoimmune or metabolic diseases result in large-scale shifts in metabolite availability, and the impact this has on how neutrophils perceive their environment and respond to infection is unclear. One major hurdle is that culture conditions routinely use media with glucose concentrations far exceeding what is typically available in the host. While one could argue that these culture conditions mimic the diabetic state, diabetes coincides with insulin resistance or lack of insulin production, which would typically limit the uptake of glucose by most cells despite the elevated concentrations. Therefore, to better understand the metabolic intricacies of neutrophils, better efforts need to be made to recreate the metabolic constraints of the host in culture systems. Furthermore, using murine models that better reflect the disease that they are supposed to mimic is essential. For example, streptozotocin has been used to cause hyperglycemia within murine models to understand immune cell function in response to infection during diabetes; however, this model does not recreate the immunologic dysregulation of T1D nor the insulin resistance observed in T2D. While the streptozotocin model is easy and relatively consistent, the goal of murine studies should be to translate these findings into human disease. Therefore, it is important not to overinterpret results and to transition towards using murine models that better reflect human disease.
